# APP Intracellular Domain Impairs Adult Neurogenesis in Transgenic Mice by Inducing Neuroinflammation

**DOI:** 10.1371/journal.pone.0011866

**Published:** 2010-07-30

**Authors:** Kaushik Ghosal, Andrea Stathopoulos, Sanjay W. Pimplikar

**Affiliations:** Department of Neurosciences, Lerner Research Institute, Cleveland Clinic, Cleveland, Ohio, United States of America; Hungarian Academy of Sciences, Hungary

## Abstract

**Background:**

A devastating aspect of Alzheimer's disease (AD) is the progressive deterioration of memory due to neuronal loss. Amyloid precursor protein (APP) occupies a central position in AD and APP-derived amyloid-β (Aβ) peptides are thought to play a pivotal role in disease pathogenesis. Nonetheless, it is becoming clear that AD etiology is highly complex and that factors other than Aβ also contribute to AD pathogenesis. APP intracellular domain (AICD) is generated together with Aβ and we recently showed that AICD transgenic mice recapitulate pathological features of AD such as tau hyperphosphorylation, memory deficits and neurodegeneration without increasing the Aβ levels. Since impaired adult neurogenesis is shown to augment memory deficits in AD mouse models, here we examined the status of adult neurogenesis in AICD transgenic mice.

**Methodology/Principal Finding:**

We previously generated transgenic mice co-expressing 59-residue long AICD fragment and its binding partner Fe65. Hippocampal progenitor cell proliferation was determined by BrdU incorporation at 1.5, 3 and 12 months of age. Only male transgenic and their respective wilt type littermate control mice were used. We find age-dependent decrease in BrdU incorporation and doublecortin-positive cells in the dentate gyrus of AICD transgenic mice suggesting impaired adult neurogenesis. This deficit resulted from decreased proliferation and survival, whereas neuronal differentiation remained unaffected. Importantly, this impairment was independent of Aβ since APP-KO mice expressing AICD also exhibit reduced neurogenesis. The defects in adult neurogenesis are prevented by long-term treatment with the non-steroidal anti-inflammatory agents ibuprofen or naproxen suggesting that neuroinflammation is critically involved in impaired adult neurogenesis in AICD transgenic mice.

**Conclusion/Significance:**

Since adult neurogenesis is crucial for spatial memory, which is particularly vulnerable in AD, these findings suggest that AICD can exacerbate memory defects in AD by impairing adult neurogenesis. Our findings further establish that AICD, in addition to Aβ, contributes to AD pathology and that neuroinflammation plays a much broader role in AD pathogenesis than previously thought.

## Introduction

Alzheimer's disease (AD) is a progressive neurodegenerative disorder characterized by a gradual decline in memory and executive functions. Mounting evidence suggests Amyloid precursor protein (APP) and presenilins are central molecules in the pathophysiology of AD [1], [2]. Aided by presenilins, APP undergoes constitutive proteolysis to produce multiple fragments including amyloid β (Aβ) peptides, which form senile plaques. A vast number of studies have shown that Aβ plays a pivotal role in the pathophysiology of AD [2] and it is thought that Aβ peptides, in an as yet uncertain form, trigger a cascade of downstream deleterious events that eventually results in AD. Aβ peptides are shown to hyperphosphorylate tau [3], induce neuronal cell death *in vitro*, and cause memory deficits in mice [4]. They are also implicated in causing silent seizures [5] and in the impairment of adult neurogenesis in AD mouse models [6]–[10].

The process of neurogenesis occurs mostly during embryogenesis but also persists during adulthood in the subventricular zone and subgranular zone (SGZ) of the hippocampus. There is increasing evidence that adult hippocampal neurogenesis plays an important role in learning and memory [11]. Increased adult neurogenesis enhances hippocampal-dependent learning and memory [12] whereas decreased neurogenesis impairs it [13]. Also, drugs that increase hippocampal neurogenesis also increase learning and memory [14] and hippocampal neurogenesis is known to decline with aging [15]. Adult neurogenesis is important for maintaining spatial memory function [13], [16], which is characteristically lost in AD patients. Thus, impaired neurogenesis is being recognized as a significant pathological feature of AD since it can further exacerbate the memory decline caused by neuronal loss.

Although, Aβ peptides have been known to play a pivotal role in AD pathology, it is becoming increasingly clear that not all aspects of AD can be accounted for by Aβ alone [17], [18]. Findings from such diverse lines of investigation as neuroimaging, clinical trials and preclinical observations suggest that non-Aβ factors also contribute to memory deficits in AD. The cleavage of APP by presenilins, which generates Aβ, also releases APP Intracellular domain (AICD) from the membrane. AICD modulates gene transcription *in vitro* [19], [20], alters signaling pathways and exerts deleterious effects in tissue culture cells [21], [22], which could cause AD-like features *in vivo*. Indeed, AICD expressing transgenic mice recapitulate such AD pathologies as activation of glycogen synthase kinase-3β (GSK3β), phosphorylation and aggregation of Tau, deficits in working memory and abnormal neural activity and silent seizures [23]–[25]. Interestingly, these pathologies were observed without appreciable changes in APP metabolism or Aβ generation [24]. Since AICD levels are elevated in human AD brains [24], these findings raise the possibility that AICD, in addition to Aβ, significantly contributes to AD pathogenesis.

In this study, we report that AICD transgenic mice also exhibit impaired adult neurogenesis by reducing neuronal proliferation and survival. This impairment is observed even in the absence of endogenous APP demonstrating that AICD alone is capable of inducing the deleterious effects. Importantly, the defective adult neurogenesis is prevented by blocking neuroinflammatory changes indicating that neuroinflammation plays a much broader role in AD pathogenesis than previously thought.

## Results

### Adult hippocampal progenitor cell (HPC) proliferation is reduced in AICD transgenic mice

**Figure 1:**
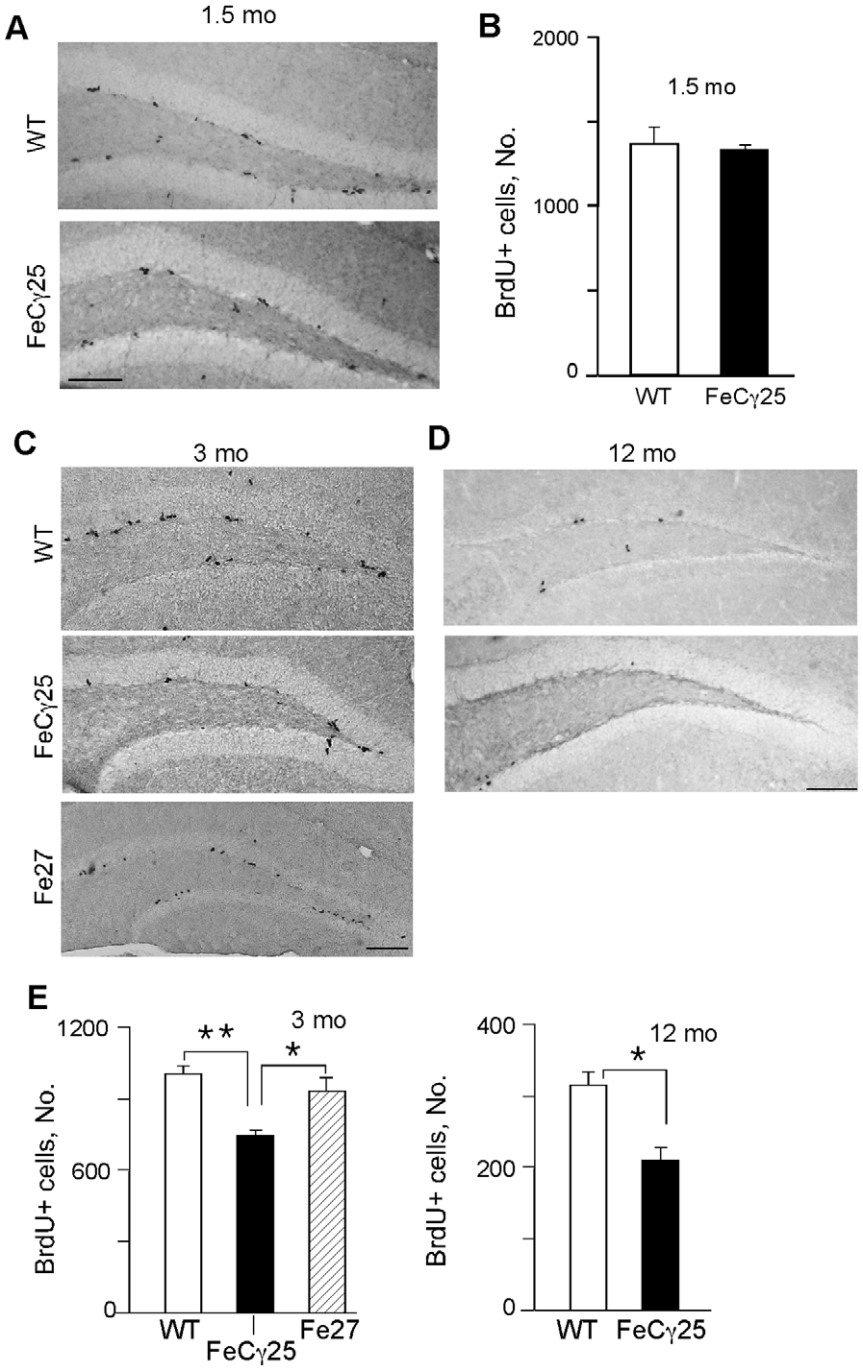
AICD transgenic mice show decreased cell proliferation in the SGZ of hippocampus. Representative images of BrdU immunostaining in the subgranular zone (SGZ) of the dentate gyrus performed on 1.5-mont-old (**A**) 3-month-old (**C**) and 12-month old (**D**) wild-type and FeCγ25 animals one day after the final BrdU injection (100 mg/kg). (**A–B**) At 1.5 mo there was no statistically significant difference in the number of BrdU-labeled cells between wild-type and FeCγ25 mice, quantified in (**B**). (**E**) Quantitative analysis of the total number of BrdU+ cells throughout the entire rostro-caudal extent of the hippocampus in 3-month-old (top) and 12-month-old (bottom) animals reveal a statistically significant decrease in the number of BrdU+ cells in the SGZ of FeCγ25 mice compared to wild-type mice. (n = 3, 4 and 4 for both wild-type and FeCγ25 mice at ages 1.5 mo, 3 mo and 12 mo respectively. n = 3 for Fe27 mice at 3 mo. * p<0.05, ** p<0.01).

We measured HPC proliferation in the SGZ by injecting male AICD transgenic mice (line FeCγ25) at 1.5, 3 or 12 months of age daily with BrdU for 3 days. Mice were sacrificed 24 hours after the last injection, brains were harvested, fixed and free-floating sections were prepared. We used male mice in this study to avoid the confounding effects of estrus cycle and gonadotrophins on adult neurogenesis in female animals [26]. Brain slices were immunostained with anti-BrdU antibody and BrdU-positive (BrdU+) cells were quantified in the SGZ (defined as the region spanning the border of granule cell layer and hilus with two cell layers into the granule cell layer and hilus) of the entire rostro-caudal extent of the hippocampus. At 1.5 months, the numbers of BrdU+ cells in FeCγ25 mice were similar to that observed in non-transgenic control littermates (Fig. 1A,B). However, 3-month-old FeCγ25 mice exhibited reduced BrdU+ cells compared to wild-type animals (Fig. 1C). Since FeCγ25 transgenic mice co-express AICD and Fe65 (which binds and stabilizes AICD), we performed an analogous experiment on Fe27 transgenic mice, which express Fe65 alone [23]. Fe27 mice showed a similar number of BrdU+ cells as the non-transgenic controls (Fig. 1C, E), indicating that AICD is required to observe impaired HPC proliferation in 3-month-old mice. The deficits in HPC proliferation were also observed in 12-month-old AICD transgenic mice (p<0.05; Fig. 1D, E). Animals older than 12 months were not studied because at this stage even the wild-type mice showed a very low level of adult neurogenesis. Together, these data suggest that AICD impairs adult HPC proliferation *in vivo*.

### Decreased hippocampal neurogenesis in AICD transgenic mice

**Figure 2:**
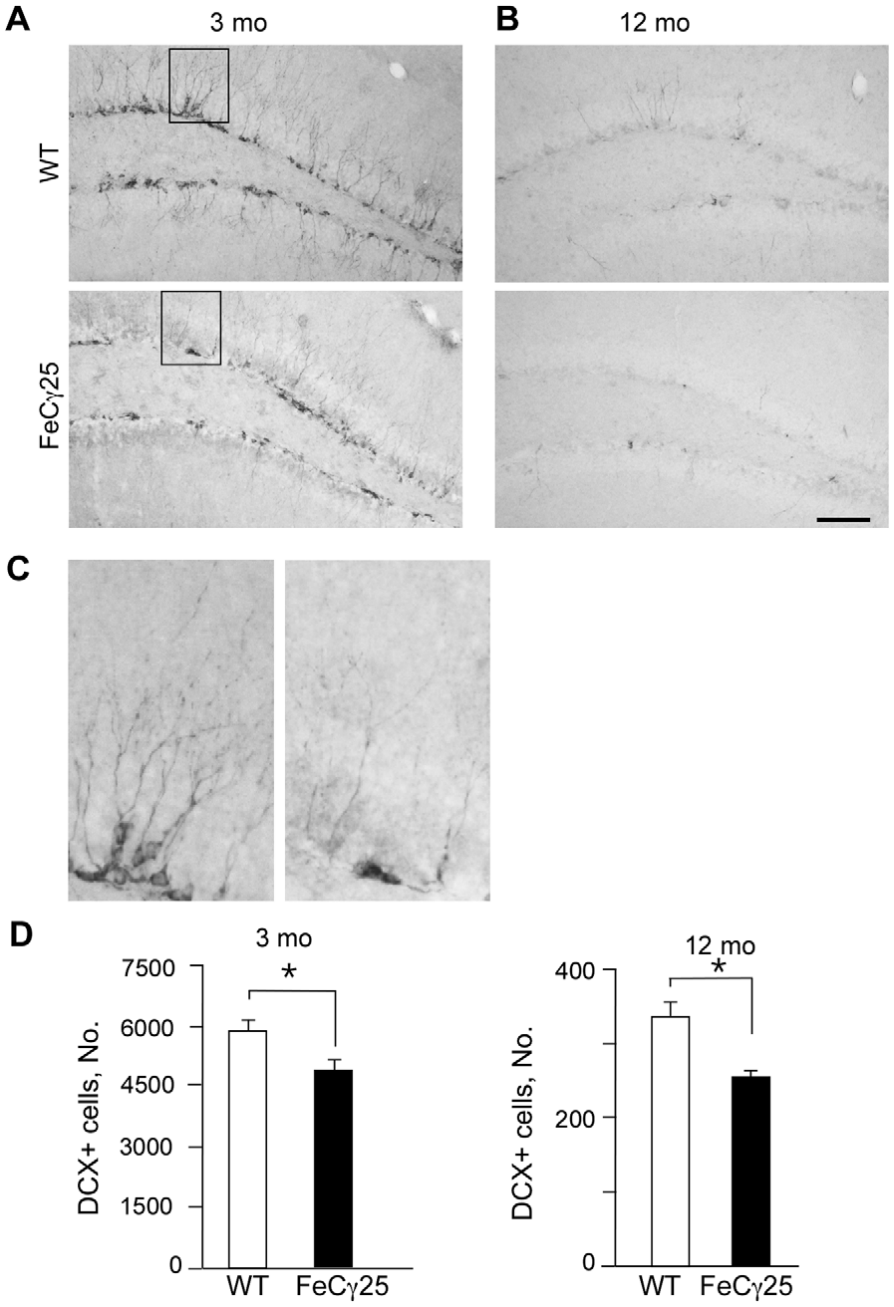
FeCγ25 mice show a decreased number of immature neurons in the SGZ of dentate gyrus (DG). **A–B,** Coronal sections from 3 mo (**A**) and 12 mo (**B**) brains of wild-type and FeCγ25 mice were immunostained for the immature neuronal marker doublecortin (DCX). There is an abundance of immature neurons in the SGZ of the granule cell layer in both the supra- and infra-pyramidal blades of the DG. Compared to the wild-type (top) there is a significant decrease in the number of DCX+ cells in the SGZ of FeCγ25 mice (bottom). **C,** Magnification of the boxed region in (**A**) show the diminished number of DCX+ cells in FeCγ25 mice at 3 mo of age with a concomitant decline in dendritic number. (**D**) Quantitative analysis of the total number of DCX+ cells throughout the entire rostro-caudal extent of the hippocampus in 3-month-old (left) and 12-month-old (right) animals reveal a statistically significant decrease in the number of DCX+ cells in the SGZ of FeCγ25 mice compared to wild-type mice. Scale bar  = 100 µm. n = 4 for all.

A majority of newborn cells in the SGZ of adult animals do not survive, and the surviving cells differentiate into neurons or glia [27]. To examine whether reduced HPC proliferation is also accompanied by reduced neurogenesis, we immunostained the brain sections with anti-doublecortin (DCX) antibody. DCX is a microtubule binding protein that is expressed by immature neurons but not by glia. At 3 months of age, wild-type mice showed a large number of highly branched DCX+ cells in the SGZ, with branch tips extending into the molecular layer (Fig. 2A). By contrast, the staining was severally diminished in age-matched FeCγ25 mice and there was a decrease in the number of DCX+ cells (Fig. 2A, 2D) as well as in the extent of arborization (see Fig. 2C). As observed above, the reduction in the number of DCX+ persisted in 12-month-old mice (Fig. 2B, 2D). Collectively, these results demonstrate that reduced HPC proliferation is accompanied by decreased neurogenesis in AICD transgenic mice and that these deficits are apparent at 3 months of age and continue to at least 12 months of age.

### AICD impairs cell survival but not cell differentiation in hippocampus

**Figure 3:**
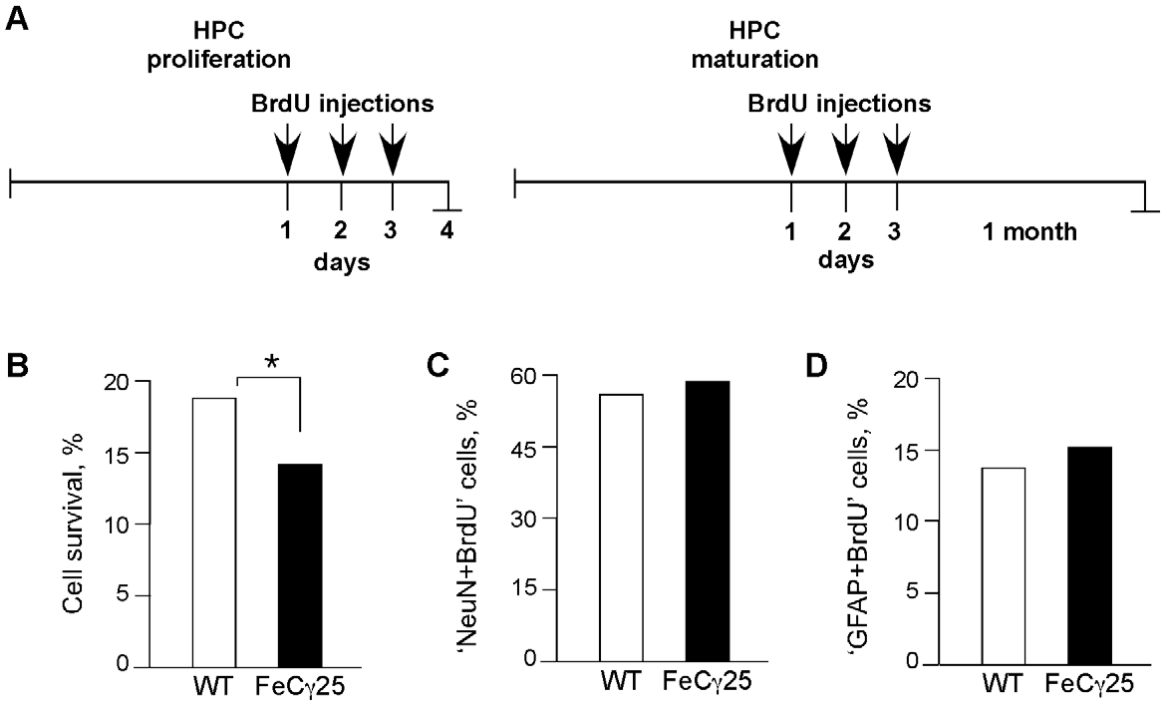
AICD downregulates cell survival but does not affect differentiation of newly generated hippocampal cells. **A,** Experimental design to measure HPC proliferation or HPC maturation. After the last BrdU injection, mice were sacrificed after 1 day (proliferation) or 1 month (maturation) and HPC survival was quantified as the ratio of the number of BrdU+ cells present at 4 weeks post-BrdU to the number of BrdU+ cells present one day after the last BrdU injection. (**B**) FeCγ25 mice (13.7, SEM 0.02) showed a significant reduction in the cell survival potential compared to wild-type mice (18.3, SEM 0.02) (* p = 0.012, χ^2^ test). **C–D,** Quantitative analysis of the number of BrdU+ cells that mature into neurons (**C**) and astrocytes (**D**) 4 weeks after birth. Analysis reveals that AICD does not influence the proportion of surviving cells that mature into neurons (NeuN+, wild-type = 54.8, SEM 0.04; FeCγ25 = 56.6, SEM 0.1) or astrocytes (GFAP+, wild-type = 14.0, SEM 0.02; FeCγ25 = 15.1, SEM 0.03). n = 3 for all.

We further corroborated the finding that AICD transgenic mice show impaired HPC proliferation by determining the cell survival and differentiation of the newborn cells. To measure cell survival, we injected mice with BrdU for three consecutive days and sacrificed them either one day or four weeks after the last injection (Fig. 3A). Most newborn granular cells die during this time period and the surviving cells differentiate into neurons or glia. Quantification of BrdU+ cells showed that survival of the newborn cells (the ratio of BrdU+ cells after 4 weeks to the BrdU+ cells after 1 day) was decreased (p<0.01) in FeCγ25 mice (Fig. 3B). Roughly 19% and 14% of newborn cells in wild-type animals and in FeCγ25 mice survived after 4 weeks respectively.

We also determined the differentiation of newborn cells into neurons and glia by performing double-immunostaining using neuronal and astrocytic markers (Fig. S1). Brain sections were incubated with anti-BrdU antibody and anti-NeuN antibody (to mark mature neurons) or anti-GFAP antibody to identify astrocytes. There were no significant differences in the percentage of ‘NeuN+BrdU’ double-labeled cells and approximately half of the newborn cells matured into neurons in both FeCγ25 and wild-type mice (Fig. 3C). Similarly, there were no significant differences in the proportion of BrdU+ cells that matured into astrocytes (Fig. 3D). Together, these results indicate that AICD decreases HPC proliferation and survival without affecting cell differentiation.

### AICD impairs HPC proliferation in mice lacking APP

**Figure 4:**
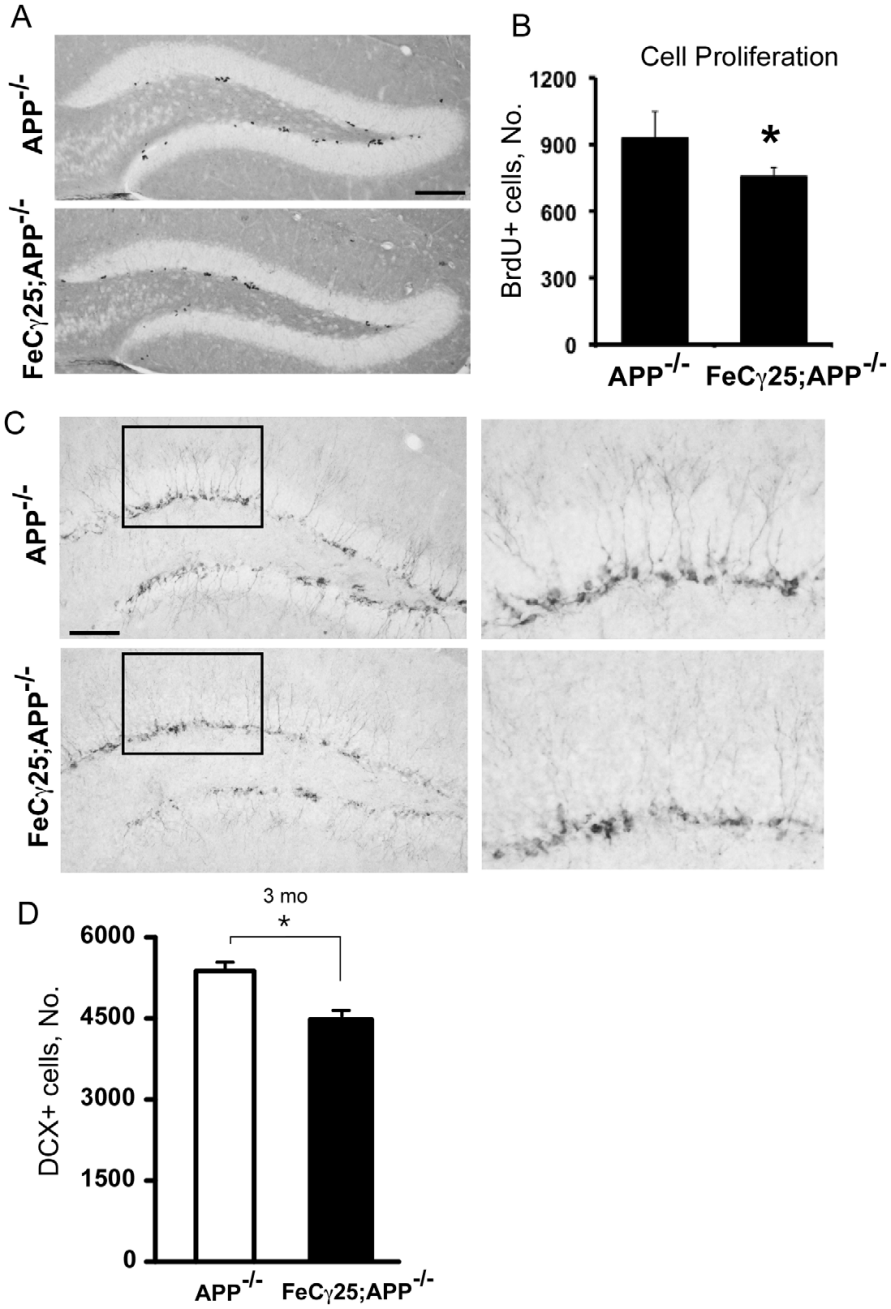
The effect of AICD is independent of other APP metabolites including Aβ. **A–B,** Decreased cell proliferation in FeCγ25;APP^−/−^ mice compared to APP^−/−^ mice. Photomicrograph reveals a reduced number of BrdU+ cells in the SGZ of FeCγ25;APP^−/−^ compared to APP^−/−^ which is quantified in (**B**) throughout the entire rostro-caudal extent of hippocampus. **C,** There was also a decrease in the number of immature neurons (DCX+) in the FeCγ25;APP^−/−^ mice compared to APP^−/−^. Insets are magnified on the right to reveal the decreased number of DCX+ cells and the resulting dendritic branches in comparable regions of the hippocampus. (**D**) Quantitative analysis of the total number of DCX+ cells throughout the entire rostro-caudal extent of the hippocampus in 3-month-old animals reveal a statistically significant decrease in the number of DCX+ cells in the SGZ of FeCγ25; APP^−/−^ mice compared to APP^−/−^mice.* p<0.05 (Student's T-test). n = 3 for all.

Adult neurogenesis is reduced in many mouse models of AD and this reduction may be caused by multiple factors [reviewed in 6]. We previously showed that APP processing and Aβ levels were unchanged in 3-month-old FeCγ25 mice compared to control mice [24]. Thus, Aβ probably plays no role in reduced adult neurogenesis in FeCγ25 animals. However, to address this issue unambiguously, we crossed FeCγ25 mice with APP knockout animals and obtained AICD-expressing APP^−/−^ mice. 3-month-old FeCγ25;APP^−/−^ mice were injected with BrdU for 3 days, brains were harvested on the 4^th^ day and sections were immunostained for BrdU (Fig. 4A, B) or DCX (Fig. 4C). The lack of APP did not seem to affect adult neurogenesis since the number of BrdU+ cells in APP^−/−^ animals (Fig. 4B) was similar to that seen in wild-type animals (see Fig. 1B). However, there was a small but significant decline (p<0.05) in the number of BrdU+ cells in the SGZ of FeCγ25;APP^−/−^ (Fig. 4 A, B). These results unambiguously show that AICD, in the absence of endogenous Aβ, can impair HPC proliferation *in vivo*. We examined the DCX+ immature neurons in the SGZ of these mice and found that FeCγ25;APP^−/−^ animals displayed a reduced number of DCX+ cells compared to APP^−/−^ mice at 3 months of age (Fig. 4C, 4D). Also, the DCX+ cells showed reduced arborization in FeCγ25;APP^−/−^ animals (Fig. 4C, right panels).

We next determined the level of HPC proliferation in two established mouse models of AD. APPPS1 mouse model expresses ‘Swedish’ mutant APP and M146L mutant presenilin [28] whereas R1.40 mice express only ‘Swedish’ mutant APP [29]. We observed a reduction in HPC proliferation in 3-month-old APPPS1 mice but not in R1.40 animals (Fig. S2) even though both mouse models show elevated levels of Aβ. We do not know whether R1.40 mice show neurogenesis defects at older ages but at a minimum, these results show that an elevation in Aβ alone does not necessarily result in reduced neurogenesis. In any case, our findings confirm the previous results of reduced adult neurogenesis in mouse AD models and extend them to show that AICD, without Aβ, is also able to impair HPC proliferation.

### Treatment with NSAIDs prevents the deficits in HPC proliferation and neurogenesis

**Figure 5:**
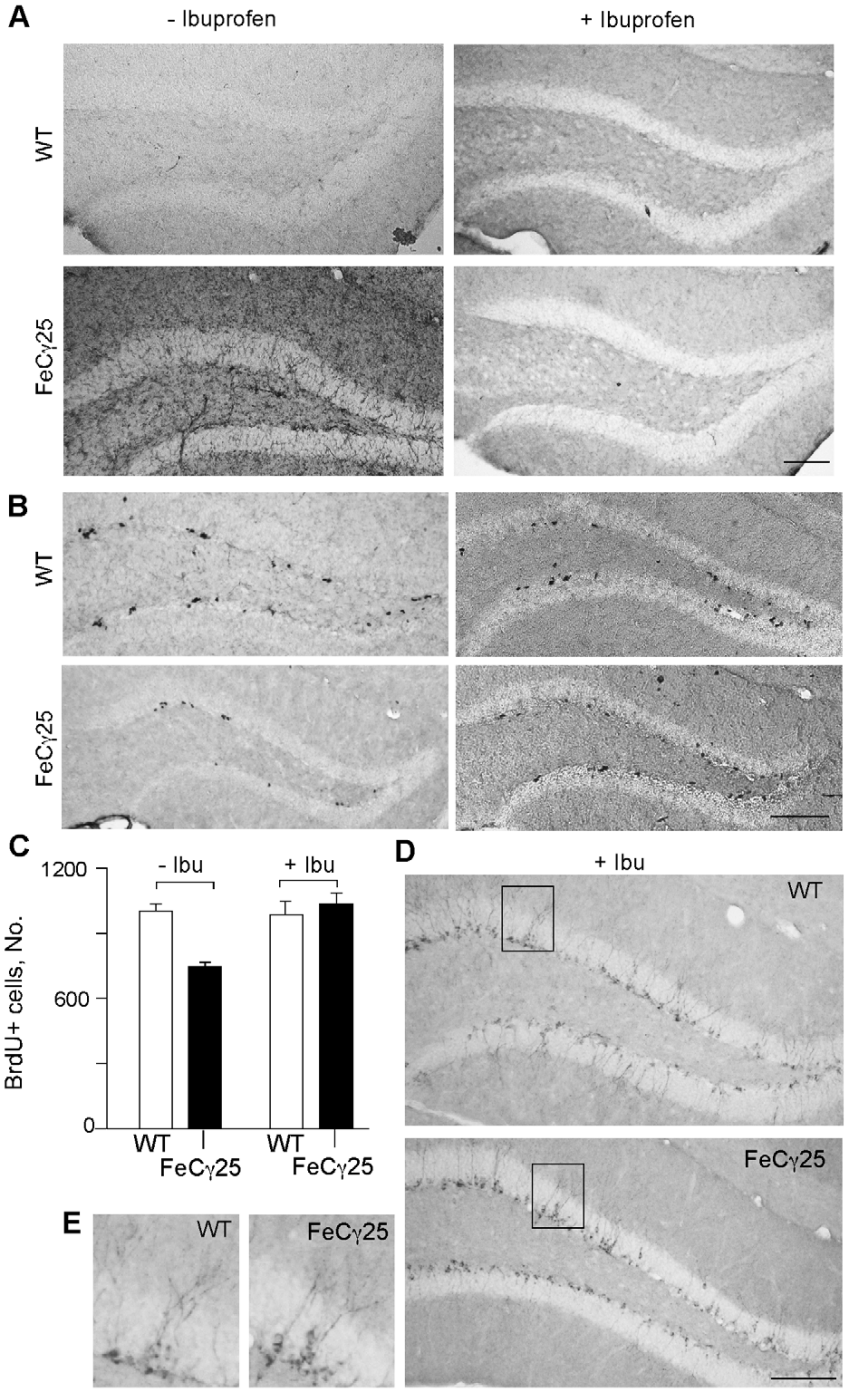
Non-steroidal anti-inflammatory drug (NSAID; ibuprofen) treatment rescued impaired adult hippocampal neurogenesis in FeCγ25 mice. **A**, CD-45 immunohistochemistry showed that ibuprofen treatment for 2 months (right) can decrease neuroinflammation seen in FeCγ25 mice compared to wild-type (WT) mice (left) at 3 months of age. **B–C**, Ibuprofen treatment rescued decreased adult hippocampal cell proliferation in FeCγ25 mice at 3 months, quantified in **C**. **D–E**, The number of immature neurons detected by doublecortin immunoreactivity was also increased in FeCγ25 mice (bottom) compared to wild-type mice (top) after ibuprofen treatment. Comparable hippocampal regions are identified in box **D** and enlarged in **E** for wild-type (left) and FeCγ25 mice (right). Error bars represent S.E.M. Scale bar  = 100 µm. n = 4 for untreated wild-type and FeCγ25 mice and n = 3 and 4 for ibuprofen treated wild-type and FeCγ25 mice respectively.

We next explored the potential mechanisms underlying the inhibitory effects of AICD. Impairment of adult neurogenesis by AICD in FeCγ25 mice is probably not mediated by cell-autonomous mechanisms because AICD expression is driven by the CaMKIIα promoter, which is active only in mature neurons [30]. However, since a number of factors including environmental stress and neuroinflammation are known to inhibit adult neurogenesis [31], [32] we determined whether FeCγ25 mice showed signs of neuroinflammation. Unexpectedly, we observed a dramatic increase in the recruitment of CD45+ microglia in 12 week-old FeCγ25 mice compared to wild-type mice (Fig. 5A). We also observed an increased expression of several proinflammatory cytokines raising the possibility that a proinflammatory environment in FeCγ25 mice was responsible for the reduction in adult neurogenesis. To test this hypothesis, we treated mice with ibuprofen, a non-steroidal anti-inflammatory drug (NSAID), by feeding animals a drug-containing diet (375 ppm) for 9 weeks beginning at 3 weeks of age. Figure 5A shows that this regimen of ibuprofen treatment completely blocked the recruitment and activation of microglia in 3-month-old mice. Concomitantly, the number of BrdU+ cells in ibuprofen-fed FeCγ25 mice increased significantly and were comparable to those seen in wild-type mice (Fig. 5B, C). Compared to FeCγ25 mice on the control diet, ibuprofen-fed FeCγ25 mice showed a 28% increase in the number of BrdU+ cells in SGZ (p<0.05). The NSAID diet did not increase the number of BrdU+ cells in wild-type mice (p = 0.31). We also performed DCX immunostaining on ibuprofen-treated animals and noticed an increase in DCX+ cells in ibuprofen-treated FeCγ25 mice (Fig. 5D). After the ibuprofen treatment, the DCX+ cells in AICD transgenic mice were morphologically indistinguishable from those in wild-type mice (Fig. 5E).

**Figure 6:**
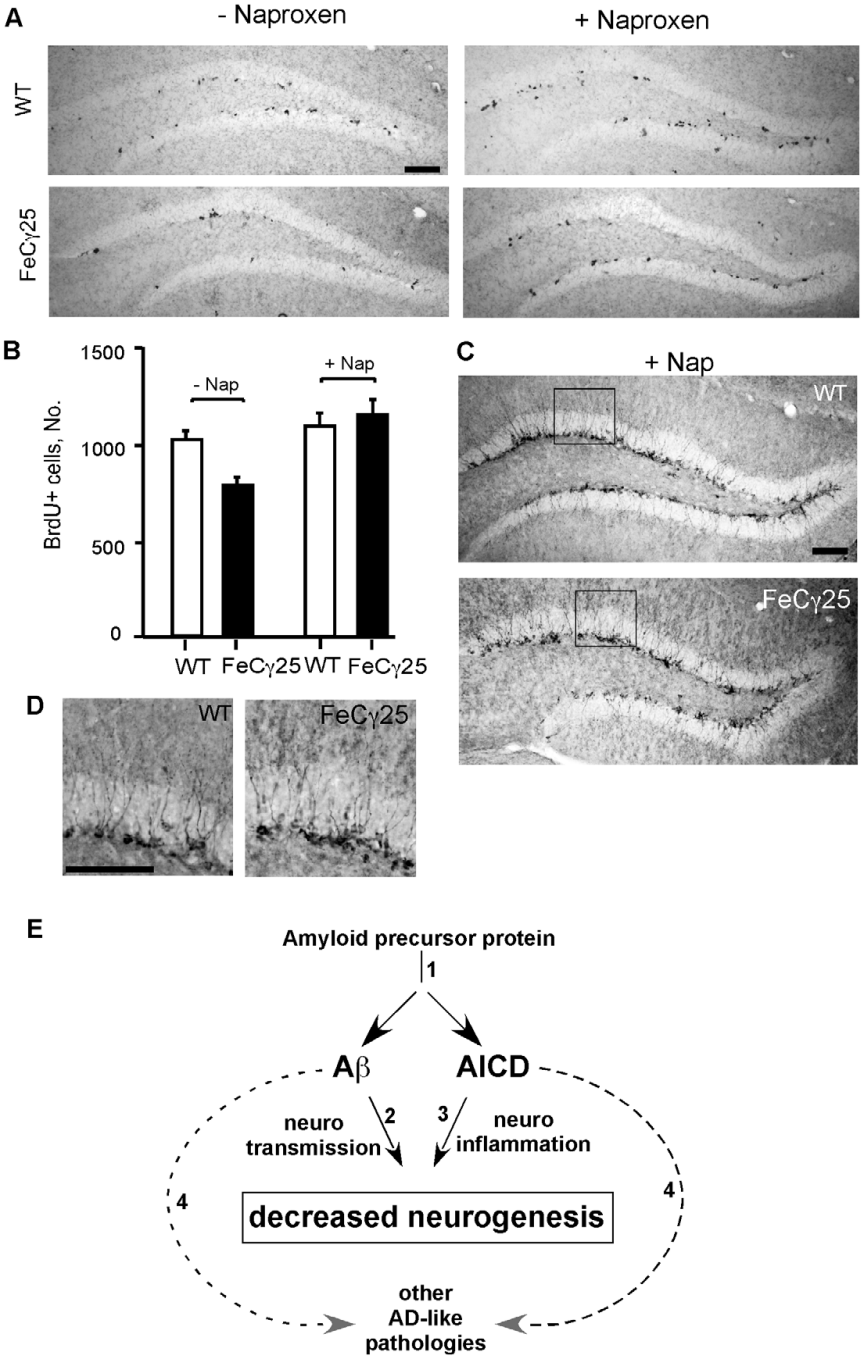
Naproxen treatment also rescued defective adult neurogenesis. **A–B,** Naproxen treatment rescued decreased adult hippocampal cell proliferation in FeCγ25 mice at 3 months, quantified in **B**. **C–D,** The number of immature neurons detected by doublecortin immunoreactivity was also increased in FeCγ25 mice (bottom) compared to wild-type mice (top) after naproxen treatment. Comparable hippocampal regions are identified in box **C** and enlarged in **D** for wild-type (left) and FeCγ25 mice (right). Error bars represent S.E.M. Scale bar  = 100 µm. n = 4 for WT, FeCγ25 and WT with naproxen and n = 6 for FeCγ25 with naproxen. **E**. A schematics of how amyloidogenic processing of APP could bring about deleterious effects via both AICD and Aβ peptides. Amyloidogenic processing [1] of APP generates Aβ and promotes AICD signaling [45]. Aβ peptides impair adult neurogenesis possibly by causing [2] an imbalance in GABAergic and Glutamatergic transmissions [10] whereas AICD does so by inducing [3] neuroinflammation (this study). Multiple studies have also shown that both Aβ and AICD peptide fragments cause [4] other AD-pathological (including tau hyperphosphorylation, neuronal cell loss, silent seizures, memory deficits) features in mice (dashed arrows).

Since ibuprofen is also known to possess off-target effects (non-COX2), we used another NSAID, naproxen, to see whether it was able to block neurogenesis defects in AICD mice. Like above, AICD mice were fed naproxen-containing diet for 9 weeks and the brains were analyzed (Figure 6 A–D). Indeed, treatment with naproxen also resulted in increased BrdU incorporation in the dentate gyrus (Figure 6A) and quantification showed that the deficits were completely rescued (Fig. 6B). Similarly, naproxen-treated mice showed increased DCX-staining that was comparable to that seen in wild-type mice (Figure 6C, D). Thus, these data establish that NSAID treatment rescues the deficits in adult neurogenesis in AICD mice by blocking neuroinflammation.

## Discussion

Here we show that AICD impairs adult hippocampal neurogenesis in an age-dependent manner. Adult neurogenesis was normal in 6-week-old AICD transgenic mice but was reduced at 3 months of age and the impairment continued until at least 12 months of age. This impairment was due to decreased cell proliferation and survival and not due to altered differentiation. The defective neurogenesis is mediated by non-cell-autonomous mechanisms involving proinflammatory changes. The important finding of this study is that impaired adult neurogenesis in AD is due to neuroinflammation and the novelty is that we provide compelling evidence that overexpression of AICD impairs adult neurogenesis without Aβ.

An increasing number of studies indicate that adult neurogenesis plays an essential role in normal memory function [11], [33]. Inhibition of adult neurogenesis impairs certain kinds of memory, such as spatial learning [13], [16]. Since the loss of spatial memory is a prominent neurological feature of AD, we suggest that impaired adult neurogenesis is especially detrimental in AD. Indeed, a vast number studies have demonstrated deficits in adult neurogenesis in amyloid-based mouse models of AD [6]–[10], [31], [34], although a conflicting study has also been reported [35]. In AICD mice, impaired neurogenesis is first observed at 3 months, which precedes memory deficits by about 4–5 months [24]. Interestingly, impaired adult neurogenesis is also detected much before the appearance of other AD-features in APPswe/PS1dE9 [9] and 3xTgAD [14] mouse models of AD. Thus, deficient adult neurogenesis is a significant pathological factor in AD and our findings suggest that therapies against AD will be more effective when supplemented with treatments that stimulate adult neurogenesis (such as exercise) or prevent impaired adult neurogenesis (such as anti-inflammatory drugs). Indeed, our preliminary studies show that NSAID treatment improves working memory deficits in the Y-maze paradigm in older (>14 month) AICD transgenic mice.

The finding that treatment with ibuprofen or naproxen prevented neurogenesis defects is of relevance to AD and supports the potential of NSAIDs as prophylactic therapeutic agents [36]. How AICD overexpression results in sustained neuroinflammation is currently under investigation but our preliminary findings suggest that AICD activates stress-kinase pathways and upregulates expression of proinflammatory cytokines. Increased levels of proinflammatory cytokine are reported in serum and CSF from AD patients [37], [38] and neuroinflammation is recognized as a significant pathological event in AD [39], [40]. A mutation in presenilin that alters APP processing inhibits adult neurogenesis *in vivo* by activating the microglia and elevating the cytokine levels [31]. Proinflammatory conditions are known to downregulate neurogenesis and blocking inflammation has been shown to restore impaired neurogenesis [32]. At present the reasons why proinflammatory environment impairs proliferation and survival more potently but leaves neuronal maturation more or less intact are not clear. Multiple studies have shown the inhibitory effects of individual inflammatory factors (such as TNF-α, IFN-γ, IL-6) on proliferation but variable effects on maturation [reviewed in 41]. NPC proliferation is orchestrated by temporal and spatial cues and involves expression of various transcription factors. It seems likely that NPC proliferation is more sensitive to the gene expression changes brought about by proinflammatory cytokines. In any case, chronic neuroinflammation in AD is likely to impair adult neurogenesis and aggravate the loss of hippocampal neurons. Interestingly, retrospective epidemiological studies [36] have shown that long-term use of NSAIDs exert a protective effect against developing AD (although short-term prospective use of NSAID was not protective) and this effect is proposed to be mediated by modulation of Aβ levels [42]. Since both drugs, ibuprofen and naproxen, protected against AICD-induced defects in adult neurogenesis, we propose that NSAIDs exert beneficial effects independent of Aβ modulation. Although our data show that inflammation plays a pivotal role in impaired neurogenesis, it is possible that AICD impairs neurogenesis also by stimulating GSK-3β activity [23]. Activation of GSK-3β alters the wnt signaling pathway and perturbs neurogenesis in mice [43], [44] and in AD patients [45].

The observation that AICD transgenic mice lacking Aβ exhibit impaired neurogenesis further strengthens the evidence that AICD also contributes to AD pathology. Although a vast amount of data support the pivotal role of Aβ peptides in AD pathogenesis [1], [2], it has become clear that AD etiology is complex and that a single causative agent cannot account for all of the available data [17], [18]. AICD causes apoptosis *in vitro* [21] and induces tau hyperphosphorylation and aggregation, aberrant neuronal activity, memory deficit and neurodegeneration *in vivo* [24], [25]. Because AICD levels are also elevated in human AD brains [24], we had hypothesized that AICD makes significant contributions to AD pathogenesis, and the present study further supports this hypothesis. Interestingly, a recent study showed that AICD signaling occurs predominantly through the amyloidogenic processing of APP [46]. This raises the possibility that the harmful effects of amyloidogenic processing are mediated by both increased Aβ as well as elevated AICD (Fig. 6E) and may explain the disappointing results from Aβ-focused clinical trials. Future studies are needed to assess the individual contribution of each peptide to AD pathogenesis.

In conclusion, our study shows that AICD transgenic mice exhibit impaired adult hippocampal neurogenesis in an Aβ-independent manner. More importantly, NSAID treatment had a protective effect against the neurogenesis defects. Together, these findings provide evidence for non-amyloid mechanisms that potentially exacerbate neuronal loss in AD and suggest that inflammation plays a much broader role in AD pathogenesis than previously appreciated.

## Methods

### Ethics Statement

All experiments were approved by the Institutional Animal Care and Use Committee of The Cleveland Clinic (Protocol Number: 08355).

### Transgenic mice

Transgenic mice co-expressing the AICD-59 and Fe65 (FeCγ25 line) or Fe65 alone (Fe27) in C57BL/6 background have been described previously [23], [24], [47]. Both transgenes are active in the brain under the control of the CAMKIIα promoter. For all experiments non-transgenic littermates or C57BL/6 mice were used as wild-type controls. APP^−/−^ have been described in detail previously [48] APPPS1 and R1.40 animals have been previously described in detail [28], [29]. To generate FeCγ25/APP^−/−^ mice that would express AICD59 and Fe65 on APP null background, FeCγ25 hemizygous mice were bred with APP^+/−^ mice to obtain FeCγ25/APP^+/−^ mice in the F1 generation. Subsequently, FeCγ25/APP^+/−^ mice were bred with APP^+/−^ to produce F2 FeCγ25/APP^−/−^, FeCγ25/APP^−/+^, APP^−/−^ and APP^+/−^ mice. All animals were housed under standard conditions with 4–5 mice per cage. Table 1 shows the various genotypes of mice used with their treatments groups.

**Table 1:** Study Design.

Genotype	Age (month)	Treatment	Test (marker)	Animals (n)
Wild Type	1.5	Regular Chow	AHP Proliferation (BrdU) Immature Neuron (DCX)	3
	3	Regular Chow	AHP Proliferation (BrdU) Immature Neuron (DCX)	4
		Regular Chow	Survival/Maturation (BrdU/NeuN/GFAP)	3
		Ibuprofen	AHP Proliferation (BrdU) Immature Neuron (DCX)	3
		Naproxen	AHP Proliferation (BrdU) Immature Neuron (DCX)	4
	12	Regular Chow	AHP Proliferation (BrdU) Immature Neuron (DCX)	4
FeCγ25	1.5	Regular Chow	AHP Proliferation (BrdU) Immature Neuron (DCX)	3
	3	Regular Chow	AHP Proliferation (BrdU) Immature Neuron (DCX)	4
		Regular Chow	Survival/Maturation (BrdU/NeuN/GFAP)	3
		Ibuprofen	AHP Proliferation (BrdU) Immature Neuron (DCX)	4
		Naproxen	AHP Proliferation (BrdU) Immature Neuron (DCX)	6
	12	Regular Chow	AHP Proliferation (BrdU) Immature Neuron (DCX)	4
Fe27	3	Regular Chow	AHP Proliferation (BrdU) Immature Neuron (DCX)	3
App−/−	3	Regular Chow	AHP Proliferation (BrdU) Immature Neuron (DCX)	3
FeCγ25;App−/−	3	Regular Chow	AHP Proliferation (BrdU) Immature Neuron (DCX)	3
APPPS1	3	Regular Chow	AHP Proliferation (BrdU)	3
R1.40	3	Regular Chow	AHP Proliferation (BrdU)	3

Age (months) represents the time when BrdU injection was carried out. All Ibuprofen and Naproxen treatments were for 9 weeks terminating at 3 months of age. n represents number of animals.

### 5-Bromo-2-deoxyuridine injection and tissue preparation

To avoid the effects of gender and gonadotropic hormones on adult neurogenesis, only male mice were used throughout the study. To determine cell proliferation, mice were injected once daily with 5-Bromo-2-deoxyuridine (BrdU; 100 mg/kg, i.p.) for three consecutive days. On the day following the last BrdU injection, animals were anesthetized and transcardially perfused with ice-cold phosphate-buffered saline (PBS) followed by PBS containing 4% paraformaldehyde. Brain were removed and fixed in PBS containing 4% paraformaldehyde overnight at 4°C, cryoprotected in 30% sucrose in PBS, embedded using OCT compound (Sakura Finetek, Torrance, CA, USA) and stored at −80°C. To determine long-term cell survival and maturation, animals were killed 28 days after the final injection of BrdU. The brains were processed as described above.

### Immunohistochemistry

Hemi-brains were fixed overnight in 4% paraformaldehyde in PBS, sunk in 30% sucrose and embedded in OCT. 30 µm sagittal sections were cut and every 12^th^ section was used. Antibodies used were BrdU (Abcam, 1∶500) and doublecortin (SantaCruz, 1∶1000). Remaining details are given in Supplemental Methods S1.

### Stereological counts

The total number of BrdU+ or DCX+ cells in the subgranular zone of the dentate gyrus was quantified using unbiased stereological methods. BrdU+ or DCX+ cells were counted from every sixth section using a 40X objective throughout the entire rostro-caudal extent of the dentate gyrus (bregma −1.0 mm to −2.80 mm). Cells were counted from both halves of the brain within the granular cell layer (GCL) and adjacent SGZ up to a two-cell body-wide zone along the border between the GCL and the hilus. The experimenter counting the cells was blinded to the genotype and experimental modification of the mice. The total number of cells was obtained by multiplying the number of BrdU+ or DCX+ cells with inter section interval and adding them together for the entire hippocampus.

### Non-steroidal anti-inflammatory drug (NSAID) diet

Ibuprofen or naproxen (both from Sigma Aldrich) was formulated into standard animal chow by Research Diets at a final concentration of 375 ppm. Male FeCγ25 and non-transgenic littermates were fed drug-supplemented or control chow for 9 weeks. Mice were killed at the end of the experimental period and processed for histology or biochemical analyses. For neurogenesis assays, animals were injected with BrdU for three consecutive days before sacrificing as described above.

### Statistics

Statistical analysis was performed using GraphPad prism 3.1. The results are expressed as mean ± standard errors of mean (SEM).

Rest of the protocols is described in Supplemental Methods (Methods S1).

## Supporting Information

Methods S1 Supplemental Methods
(0.03 MB DOC)

Figure S1 Differentiation of newborn granular cells into neurons and astrocytes. Mice were injected with BrdU and sacrificed one month after the last injection. Brains were harvested and free-floating sections were incubated with anti-BrdU antibody with anti-NeuN antibody (A) or anti-GFAP antibody (B). Confocal image analysis was used to score the coexpression of BrdU (green) with the neuronal marker NeuN (A, red) or the astrocyte marker GFAP (B, red).
(6.99 MB TIF)

Figure S2 Decreased neurogenesis in other mouse models of AD. A, Thioflavin-S staining of 3-month-old animals showed increased plaque density in APPPS1 animals but a complete absence in FeCγ25 transgenic animals. B, BrdU immunostaining on 3-month-old animals showed decreased cell proliferation in APPPS1 mice similar to FeCγ25 mice. R1.40 animals do not show a decline and behaved similar to WT animals. C, Quantification of BrdU counts throughout the entire hippocampus revealed a significant decrease in cell proliferation for both FeCγ25 and APPPS1 animals compared to R1.40 and WT mice. ^*^ p<0.05 ANOVA. N = 4 for WT and FeCγ25 and 3 for APPPS1 and R1.40. Scale bar  = 100 µm
(3.53 MB TIF)
