# Supplementary material for: APP Intracellular Domain Impairs Adult Neurogenesis in Transgenic Mice by Inducing Neuroinflammation
Source: PLoS One. 2010 Jul 30;5(7):e11866. doi: 10.1371/journal.pone.0011866 (PMC2912762; doi:10.1371/journal.pone.0011866)
Supplement: Methods S1 — Supplemental Methods (0.03 MB DOC) [file pone.0011866.s001.doc]

*Immunohistochemistry*

Coronal sections were cut at a 30 μm thickness and stored free-floating in cryo-storage solution (30% ethylene glycol, 30% sucrose, 1% PVP-40 in 0.2M Sorenson's buffer). For single labeling of BrdU, free-floating sections were rinsed extensively in PBS and endogenous peroxidase activity was quenched by incubating the sections in 3% H_2_O_2_ in PBS containing 0.1% Triton-X (PBST) for 30 min. After thorough washing, sectioned were incubated in 2N HCl to denature DNA for 2 hr at room temperature. For doublecortin (DCX) immunohistochemistry, sections were rinsed in PBS and incubated in 3% H_2_O_2_ in PBS containing 0.1% PBST for 30 min to quench endogenous peroxidase activity. After thorough washes in PBS, sections were blocked in PBST containing 5% normal goat serum for 1 hr for BrdU immunohistochemistry or normal donkey serum for DCX immunohistochemistry, followed by incubation in monoclonal rat anti-BrdU antibody (1:500, AbCam, USA) or goat polyclonal anti-DCX (1:1000, Santa Cruz, Santa Cruz, CA, USA) overnight at 4^0^C. The following day, sections were rinsed several times in PBST and incubated in appropriate biotinylated secondary antiserum (1:200, Vector Laboratories) for 2 hr at room temperature. Following incubation and subsequent washes, sections were incubated for 90 min at room temperature in avidin–biotin–peroxidase complex (Vectastain ABC kit; Vector Laboratories, Burlingame, CA, USA). Staining was developed at room temperature in PBS containing 0.25 mg/ml 3,3'-diaminobenzindine-HCl (DAB; Sigma-Aldrich, St Louis, Missouri, USA) and 0.03% H_2_O_2_. The reaction was stopped by rinses in PBS. Sections were mounted using glycerol and placed under a coverslip. Microscopy was performed using a Leica DMR microscope equipped with a CCD camera for bright field imaging.

*Double immunofluorescence detection of cell fate*

Immunostaining for confocal analysis was performed on 30 µm coronal brain sections. These sections were used for double-immunofluorescence labeling of BrdU with markers of postmitotic neurons (NeuN) and astrocytes (glial fibrillary acidic protein or GFAP). Sections were washed in PBS and denatured with 2N HCl as described above, followed by blocking in 5% normal donkey serum in PBST for 1 hr at room temperature. Sections were then incubated overnight at room temperature in a mixture of monoclonal rat anti-BrdU antibody (1:500) and monoclonal mouse anti-NeuN (1:1000, MAB377; Chemicon, Temecula, CA) or rabbit anti-GFAP (1:1000, Dako, Carpinteria, CA, USA) in PBST. Following several rinses, the sections were incubated for 2 hr at room temperature in secondary antibodies Alexa 488 goat anti-rat IgG (1:250; Invitrogen, Carlsbad, CA) and Alexa 594 goat anti-mouse IgG (1:250; Invitrogen) or Alexa 555 goat anti-rabbit IgG (1:250; Invitrogen) in PBST. After rinsing out the excess antibody, sections were mounted on glass slides using Vectashield (Vector Laboratories) containing DAPI and were stored at 4°C. Sections were imaged using a Leica Confocal SP5 microscope (Leica Inc., USA).

*Thioflavin-S staining*

Free floating brain sections were mounted on slides and washed in PBS for 30 min. Following PBS wash, sections were incubated in 1% Thioflavin-S for 15 min. After washing out excess Thioflavin-S in PBS, sections were placed under a coverslip and mounted in 50% glycerol in PBS. Images were taken using a fluorescence microscope.
