# Supplementary figures and images for: APP Intracellular Domain Impairs Adult Neurogenesis in Transgenic Mice by Inducing Neuroinflammation
Source: PLoS One. 2010 Jul 30;5(7):e11866. doi: 10.1371/journal.pone.0011866 (PMC2912762; doi:10.1371/journal.pone.0011866)

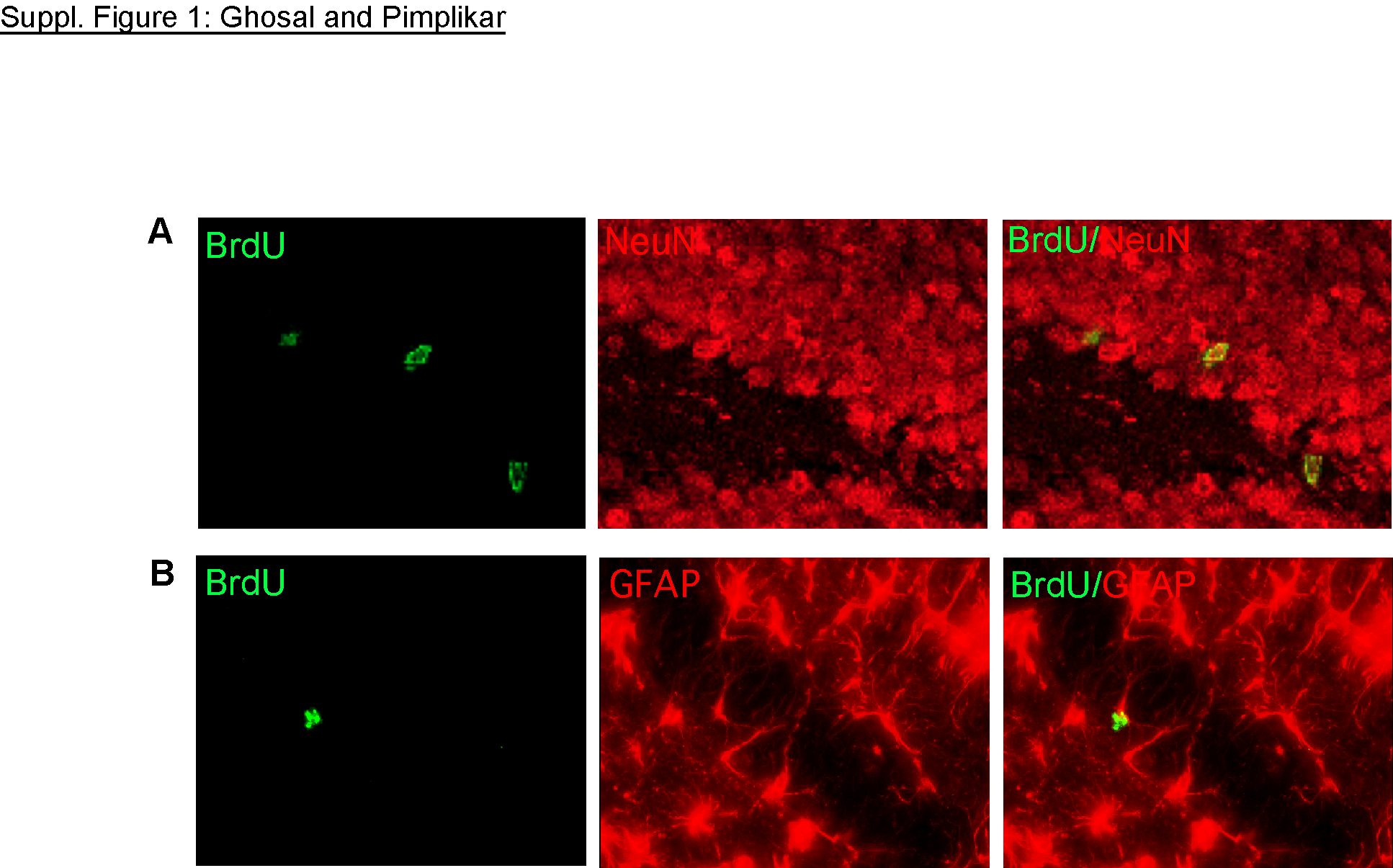

Supplement: Figure S1 — Differentiation of newborn granular cells into neurons and astrocytes. Mice were injected with BrdU and sacrificed one month after the last injection. Brains were harvested and free-floating sections were incubated with anti-BrdU antibody with anti-NeuN antibody (A) or anti-GFAP antibody (B). Confocal image analysis was used to score the coexpression of BrdU (green) with the neuronal marker NeuN (A, red) or the astrocyte marker GFAP (B, red). (6.99 MB TIF) [file pone.0011866.s002.tif]

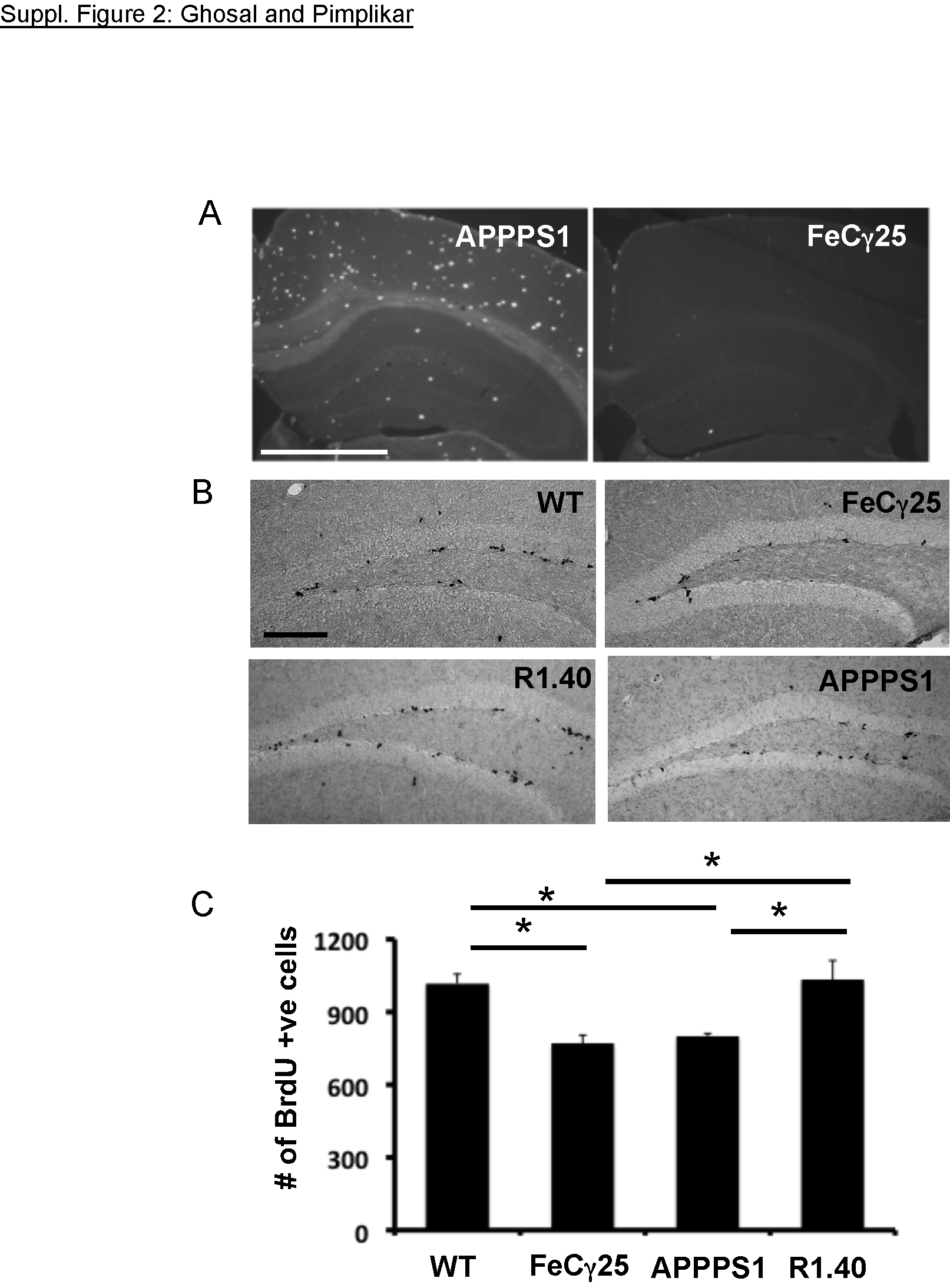

Supplement: Figure S2 — Decreased neurogenesis in other mouse models of AD. A, Thioflavin-S staining of 3-month-old animals showed increased plaque density in APPPS1 animals but a complete absence in FeCγ25 transgenic animals. B, BrdU immunostaining on 3-month-old animals showed decreased cell proliferation in APPPS1 mice similar to FeCγ25 mice. R1.40 animals do not show a decline and behaved similar to WT animals. C, Quantification of BrdU counts throughout the entire hippocampus revealed a significant decrease in cell proliferation for both FeCγ25 and APPPS1 animals compared to R1.40 and WT mice. * p<0.05 ANOVA. N = 4 for WT and FeCγ25 and 3 for APPPS1 and R1.40. Scale bar = 100 µm (3.53 MB TIF) [file pone.0011866.s003.tif]
